# Improving Mental Health Literacy and Reducing Psychological Problems Among Teachers in Zambia: Protocol for Implementation and Evaluation of a Wellness4Teachers Email Messaging Program

**DOI:** 10.2196/44370

**Published:** 2023-03-06

**Authors:** Belinda Agyapong, Charles Chishimba, Yifeng Wei, Raquel da Luz Dias, Ejemai Eboreime, Eleanor Msidi, Syed Sibte Raza Abidi, Maryn Mutaka-Loongo, James Mwansa, Rita Orji, John Mathias Zulu, Vincent Israel Opoku Agyapong

**Affiliations:** 1 Department of Psychiatry University of Alberta Edmonton, AB Canada; 2 Lusaka Apex Medical University Lusaka Zambia; 3 Department of Psychiatry Faculty of Medicine Dalhousie University Halifax, NS Canada; 4 Faculty of Computer Sciences Dalhousie University Halifax, NS Canada

**Keywords:** burnout, stress, Zambia, Africa, teacher, educator, school, anxiety, wellness, depression, e-mental health, intervention, health literacy, mental health, depressive, psychological issue, psychological problem, text message, messaging, decision-making

## Abstract

**Background:**

Primary, basic, secondary, and high school teachers are constantly faced with increased work stressors that can result in psychological health challenges such as burnout, anxiety, and depression, and in some cases, physical health problems. It is presently unknown what the mental health literacy levels are or the prevalence and correlates of psychological issues among teachers in Zambia. It is also unknown if an email mental messaging program (Wellness4Teachers) would effectively reduce burnout and associated psychological problems and improve mental health literacy among teachers.

**Objective:**

The primary objectives of this study are to determine if daily supportive email messages plus weekly mental health literacy information delivered via email can help improve mental health literacy and reduce the prevalence of moderate to high stress symptoms, burnout, moderate to high anxiety symptoms, moderate to high depression symptoms, and low resilience among school teachers in Zambia. The secondary objectives of this study are to evaluate the baseline prevalence and correlates of moderate to high stress, burnout, moderate to high anxiety, moderate to high depression, and low resilience among school teachers in Zambia.

**Methods:**

This is a quantitative longitudinal and cross-sessional study. Data will be collected at the baseline (the onset of the program), 6 weeks, 3 months, 6 months (the program midpoint), and 12 months (the end point) using web-based surveys. Individual teachers will subscribe by accepting an invitation to do so from the Lusaka Apex Medical University organizational account on the ResilienceNHope web-based application. Data will be analyzed using SPSS version 25 with descriptive and inferential statistics. Outcome measures will be evaluated using standardized rating scales.

**Results:**

The Wellness4Teachers email program is expected to improve the participating teachers’ mental health literacy and well-being. It is anticipated that the prevalence of stress, burnout, anxiety, depression, and low resilience among teachers in Zambia will be similar to those reported in other jurisdictions. In addition, it is expected that demographic, socioeconomic, and organizational factors, class size, and grade teaching will be associated with burnout and other psychological disorders among teachers, as indicated in the literature. Results are expected 2 years after the program’s launch.

**Conclusions:**

The Wellness4Teachers email program will provide essential insight into the prevalence and correlates of psychological problems among teachers in Zambia and the program’s impact on subscribers’ mental health literacy and well-being. The outcome of this study will help inform policy and decision-making regarding psychological interventions for teachers in Zambia.

**International Registered Report Identifier (IRRID):**

PRR1-10.2196/44370

## Introduction

### Background

Teaching is a high-demanding profession, and teachers experience a high prevalence of stress, burnout, anxiety, and depression. In a study, 25.3% of teachers reported mild to moderate stress [1], and high burnout was reported by 33.3% of teachers, followed by 27.6% at risk for moderate burnout [2]. In addition, 38% of teachers reported clinically significant anxiety [3], while depressive symptoms were also found in 35.3% of the teachers in a study [4]. Burnout, also known as professional exhaustion syndrome, is characterized by extreme and prolonged or recurrent stress at work [5-7]. The prevalence of burnout ranged from 25.12% to 74%, stress ranged from 8.3% to 87.1%, anxiety ranged from 38% to 41.2%, and depression ranged from 4% to 77% when only clinically meaningful (moderate to severe) psychological conditions were considered among teachers [8]. When homeostasis is disturbed, stress is produced. According to the stress framework system, there are 3 kinds of stress: sustress (inadequate stress), eustress (good stress), and distress (bad stress) [9]. Eustress may have health benefits, but both sustress and distress may lead to the impairment of normal physiological functions and could result in pathological conditions [9]. Burnout among professionals such as teachers is a stress-related problem and a risk factor for depression and other cardiovascular diseases [10,11]. According to Gluschkoff, school-related stress may lead to depressive symptoms among teachers [12,13], and often, teachers feeling more stressed are also burned out [14]. Various studies have also reported that the type of school, income satisfaction, depression, and perceived stress were all significantly associated with burnout [15,16]. Stress and emotional demands linked with teaching can lead to emotional exhaustion and lower job satisfaction [17]. Symptoms of stress and burnout among teachers directly or indirectly affect their teaching and students’ performance [17,18].

Literature has reported several predictors of teacher stress, burnout, anxiety, and depression, including age, job satisfaction, subject taught, and work-related factors [8,16,19-23]. Teachers constantly face increased challenges such as workload, role conflict, and role ambiguity, which cause work stressors and result in mental and physical health challenges [24]. Role conflict and role ambiguity also predict teachers’ burnout [25]. Teachers’ stress levels increase when their demand exceeds their capability [26-28], leading to burnout and anxiety [29,30]. Several studies have reported a significant overlap between stress, burnout, anxiety, and depression [31-36]. A survey by Besse et al [37] reported that teachers with moderate depressive disorder had higher levels of perceived stress, anxiety, low job satisfaction, and a relatively lower quality of life. Depression among teachers can also significantly affect their health, productivity, and functionality and it typically has persistent effects in both their personal and professional lives [37,38]. Teachers with low job satisfaction are also more susceptible to experiencing burnout, high anxiety levels, and depression [2,39].

Teachers in African countries similarly experience stress, burnout, depression, and anxiety. A cross-sectional study in Egypt by Desouky and Allam [20] reported a high prevalence of occupational stress (100%), anxiety (67.5%), and depression (23.2%) among teachers. This study also indicated that workload had a positive relationship with stress and that occupational stress scores were significantly higher among teachers with an increased workload [20]. A study in Ghana also reported that a poor workplace environment was associated with increased anxiety and depressive symptoms [40]. In Lusaka, Zambia, a teacher survey said that 81% of the participants rated stress levels as often, and 59% of the respondents acknowledged experiencing health-related problems due to stress [18]. Several studies also indicate that sources of stress among teachers include low salaries or income, work-related issues, a heavy workload, poor school climate, and large class sizes [1,18,24]. Another study in Zambia also reported that teachers moderately experienced stress that manifests emotionally in terms of feelings of fatigue, cardiovascular manifestations, and gastronomic manifestations [41]. These work-related psychological problems teachers encounter need to be addressed at institutional and professional levels. One approach to address the above-noted psychological issues is directly providing teachers with mental health support through educational programs that improve their mental health literacy. Teachers need to obtain and sustain positive mental health themselves. They have the skills to decrease the stigma associated with mental health problems and to promote and encourage students to seek timely and appropriate help when necessary [42]. Moreover, teachers play an essential role as partners in identifying, preventing, and intervening in mental health problems among children and youths [43,44]. Hence, improving teachers’ mental health literacy will benefit teachers and students.

Health literacy has recently transformed into a broader concept fundamental for improving an individual’s health outcome, reducing health inequities in populations, and enhancing the operation and development of health systems and policies [42,45,46]. Mental health literacy, in particular, plays a significant role in improving access to mental health care and reducing stigma related to mental problems [47]. Studies have shown that individuals with strong health literacy skills have better health and well-being than those with weak skills [45]. A survey by Kutcher [48] in sub-Saharan Africa noted that there are high stigma levels, poor mental health literacy, and less capacity at the community level to address this health care need. In another study, Kutcher [46] suggested that mental health literacy interventions should be appropriate and delivered within educational settings using modern electronic delivery platforms and strategies that enhance literacy competencies.

In Zambia, the number of frontline mental health workers, psychologists, and occupational therapists is limited and has been declining over time [49]. This has created mental health literacy and psychological treatment gaps that impact teachers and the public. Innovative, human resource-independent and cost-effective ways of delivering mental health literacy and psychological interventions to teachers in Zambia and other low-resource countries, particularly in sub-Saharan Africa, are urgently needed. The use of digital technology–based services such as SMS text and email messaging is an innovative and practical way to deliver mental health intervention and programs to improve access to mental health education and psychological help [50]. Cognitive behavioral therapy–inspired daily supportive messages delivered via SMS text messaging and web-based applications were effectively used to support population-level mental health in Canada during the COVID-19 pandemic and helped to reduce stress, anxiety, and depression [51,52]. Email messaging is another accessible and unique way to deliver low-cost cognitive behavioral therapy–based psychological interventions to the general public with mental health problems [50,53]. Participants’ responses on the satisfaction survey associated with the use of supportive SMS text messages in Canada endorsed their desire to receive the same intervention in the form of email [54].

There are about 7 billion mobile phone users globally, and developing countries account for 80% of newly purchased mobile phone devices. Therefore, mobile phone access and ownership are on the rise worldwide, and the advancement in mobile technology has helped shape digital communication [55]. Currently, most mobile phones allow for data plans and internet Wi-Fi connectivity, which makes it possible for individuals to access their emails on their cell phones. Thus, email technology may be easily accessible to teachers with mobile phones who can readily read emails on these devices. Furthermore, teachers who do not have mobile phones can still access emails through personal, institutional, or public computers, making this intervention widely available to all teachers. A study by Shalaby et al [54] also reported that 60% of respondents agreed to receive email messages as part of their health care, an indication of the high level of acceptability of using email as an eHealth tool.

According to Mayeya [49], the mental health services situation in Zambia could be considered critical, requiring immediate attention. To the best of our knowledge, no previous studies have been conducted to assess teachers’ mental health literacy levels in Zambia. For this study, 2 cities along the rail line (Livingstone and Lusaka) and 2 cities away from the rail line (Kasama and Solwezi) would be chosen. This would ensure comprehensive and acceptable findings for all teachers’ mental wellness in Zambia. A supportive email messaging program for teachers in Zambia seems timely. It can reduce the gaps in psychological interventions available to teachers and improve their mental health literacy.

### Study Objectives, Aims, and Purpose

The primary objective of this study is to determine how daily supportive email messages plus weekly mental health literacy information delivered via email would help improve mental health literacy and reduce the prevalence of moderate to high stress symptoms, burnout, moderate to high anxiety symptoms, moderate to high depression symptoms, and low resilience among schoolteachers in Zambia. The secondary objectives of this study are to map and determine the baseline prevalence and correlates of moderate to high stress, burnout, moderate to high anxiety, moderate to high depression, and low resilience among schoolteachers in Zambia. The specific objectives are as follows: (1) to determine the baseline prevalence and correlates of moderate to high stress, burnout, moderate to high anxiety, moderate to high depression, and low resilience among school teachers in Zambia. (2) To implement daily supportive email messages and weekly mental health literacy information delivered via email to improve mental health literacy. (3) To determine the effectiveness of implementing the Wellness4Teachers email messaging and mental health literacy program to reduce the prevalence of moderate to high stress symptoms, burnout, moderate to high anxiety symptoms, moderate to high depression symptoms, and low resilience among school teachers in Zambia.

**Figure 1:**
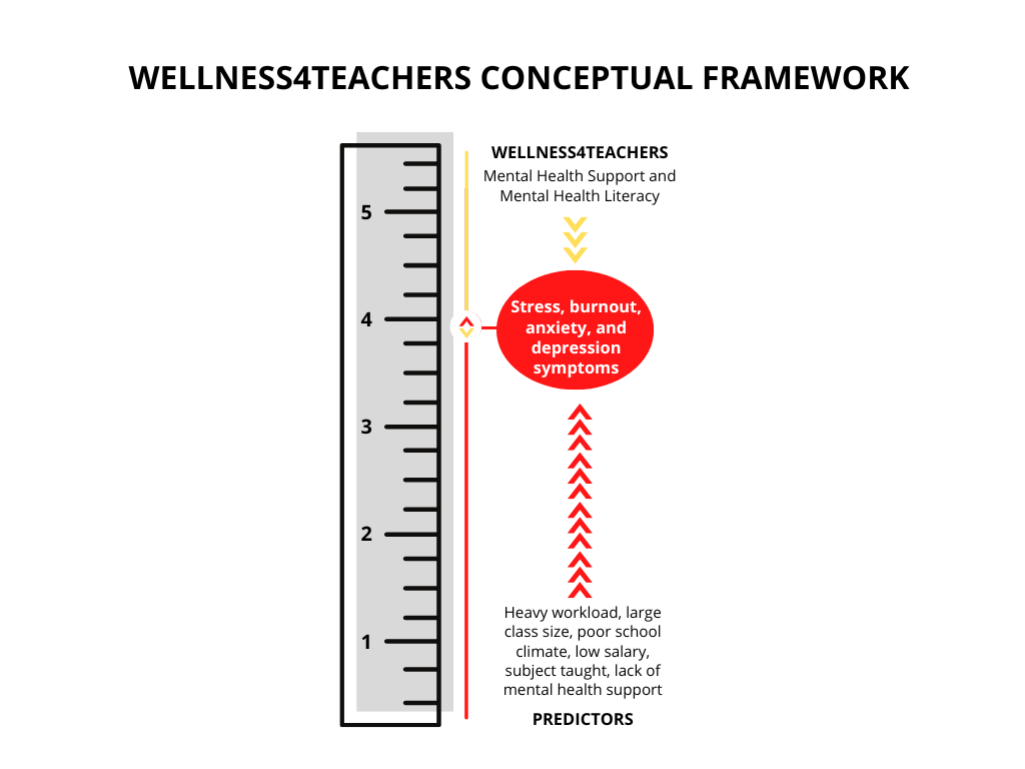
Conceptual framework.

Wellness4Teachers email messaging and mental health literacy program is anticipated to reduce the prevalence and severity of stress, burnout, anxiety, depression, and low resilience symptoms among Zambian teachers by at least 20%. Figure 1 is a conceptual framework illustrating the association between the various sociodemographic, personal, and work-related or organizational factors and stress, burnout, anxiety, and depression in teachers. The framework was based on empirical evidence, which showed a greater than 20% reduction in depression symptoms in 2 randomized controlled trials [56,57] and a greater than 20% reduction in anxiety symptom scores from baseline to 6 weeks and 3 months in subscribers of Text4Hope [51,52]. An increased number of predictors such as socioeconomic and demographic factors, workload, and poor organizational structure may correlate with the prevalence and severity of burnout and other psychological and mental health conditions experienced by teachers [3,16,19-23,49,58].

### Wellness4Teachers Email Messaging Program

The Wellness4Teachers email messaging program is one of the many suites of ResilienceNHope messaging programs, powered by the ResilienceNHope web-based application [53,59] and facilitated by the Global Psychological eHealth Foundation [60]. Wellness4Teachers email program is a daily supportive email messaging program designed to address stress, burnout, anxiety, and depression. The messages have been crafted by mental health therapists, psychiatrists, and psychologists based on the principles of cognitive behavioral therapy and have also been reviewed by the lead author, an education specialist. Teachers can personally subscribe to the Wellness4Teachers email program through the Lusaka Apex Medical University institutional account through collaboration between the Lusaka Apex Medical University and the local teachers’ unions. Teachers will receive daily supportive messages designed to address stress, burnout, anxiety, and depression and weekly links to mental health literacy information for 1 year. The email messages will be noninteractive (one-way), and subscribers will be informed of the noninteractive nature of the email messaging program during the initial welcoming and introductory message. Different messages will be received daily by subscribers from a bank of messages. The mental health crisis services number in Livingstone, Lusaka, Kasama, and Solwezi will be included in the introductory message to subscribers, which they can call if they are in crisis. Examples of messages to be delivered to subscribers of the Wellness4Teachers email messaging program include:

- Even though thoughts are often seen as accurate, they are just guesses, not facts. Burnout and stress can make you experience negative emotions, making your thoughts overly negative. Take a moment to notice how you feel. Try to replace negative thoughts with positive thoughts and actions.
- Effective goals are “SMART”: Specific, Measurable, Achievable, Realistic, and Timely. Think of one thing you want to accomplish. Break it down into smaller steps. Only focus on one small step, not the big overwhelming goal. General goals (eg, Getting fit) are not motivating. Making goals more specific (eg, Walk twice this week) are more achievable.
- We each have different signature strengths. It's not about competing with others but learning our unique strengths. Strengths come in many varieties: humor, curiosity, persistence, kindness, love, forgiveness, cooperativeness, and optimism. Share your strength with others. Help a struggling colleague.

Examples of the weekly mental health literacy information sent to subscribers can be found on the Global Psychological eHealth Foundation and the Mental Health Literacy for Educators websites [60,61].

ResilienceNHope messaging programs are evidence-based e-mental health programs designed to address mental health literacy and close the psychological treatment gap for individuals and communities globally [59]. The efficacy of the ResilienceNHope suite of programs has been evaluated and established through several randomized controlled clinical trials and evaluations of population-level programs. In a randomized controlled trial in Fort McMurray, Alberta, Canada, involving 73 patients diagnosed with major depressive disorder, the change in the mean difference in the Beck Depression Inventory scores between the intervention group (those who received twice-daily supportive SMS text messages for 3 months) and the control group (those who received a single thank-you message) was significant with an effect size (Cohen *d*) of 0.67 [62]. A similar trial was conducted in Ireland with similar results [63,64]. In addition, there were statistically significant reductions in the prevalence and mean scores on standardized measures for stress, anxiety, and depression at 6 weeks and 3 months for subscribers to the Text4Hope program [51,52].

## Methods

### Study Design

This will be an interventional study, specifically a quasi-experimental design, which will employ a quantitative longitudinal and cross-sectional survey methodology with data collected from subscribers of Wellness4Teachers email messaging service users.

### Data Collection

Quantitative data will be collected using web-based questionnaires through the ResilienceNHope secure platform for managing web-based surveys and databases [57]. The web-based questionnaires will be designed to collect demographic and work-related variables and assess mental health literacy and clinical variables, including stress, burnout, anxiety, depression, and resilience [59]. The web-based surveys will be distributed to subscribers on enrollment at 6 weeks, 3 months, 6 months, and 12 months. The follow-up surveys will also include questions assessing subscriber experience and satisfaction with the Wellness4Teachers email messaging program.

### Participants’ Recruitment and Subscription

Subscription to the Wellness4Teachers email messaging program will be initiated by individual teachers accepting an invitation to subscribe from the Lusaka Apex Medical University’s organizational account on the ResilienceNHope web-based application. The local principal investigator (CC) will work with the Zambian Ministry of Education, the Lusaka Teachers Association, district school boards, and heads of elementary and high schools to invite teachers to join the Wellness4Teachers email messaging program. Participants will be eligible for inclusion if they are school teachers and residents of Livingstone, Lusaka, Kasama, or Solwezi. Subscribers can unsubscribe from the program at any time by clicking the unsubscribe link associated with the messages. Based on the 10% dropout rate recorded for the Text4Hope program [51,52], the anticipated dropout rate for the email messaging program will be less than 15% [54].

### Study Setting

The study will occur in Zambia. Zambia is an eastern, sub-Saharan African country with an estimated 2021 population of 18,920,657 and nearly half of the population under the age of 15 years, with almost equal proportions of males and females (49.5% and 50.5%, respectively) [65]. Zambia covers about 290,587 square miles (743,390 km^2^), and the population’s median age is only 16.8 years [65,66]. The country is prone to natural and man-made disasters such as floods, droughts, mine accidents, and deforestation [49]. War and other political uncertainty in surrounding states result in a refugee influx. The study will focus on 4 main cities in Zambia: Livingstone, Lusaka, Kasama, and Solwezi. Livingstone is a capital city in the southern part of the country, with a land area of 85,283 km^2^ and a population of 1,799,885 in 2014. There were about 1241 basic schools in 2012 and 45 high schools in 2004. In 2014, there were 6158 teachers in school grades 1-9, and 1027 in high school [67].

Lusaka is the capital of Zambia and one of the 20 cities in Zambia, with a total land area of 375 km^2^ with an approximate population of 3,042,000 in 2022, a 4.68% increase from 2021 [68]. There were 111 secondary schools and 758 primary schools in Lusaka in 2017 [66], and about 10,822 primary school teachers and 3383 secondary school teachers in Lusaka in 2016 [66]. There are several rural cities in Zambia, including Kasama (Northern Province) and Solwezi (North Western Province). Kasama had a land area of 77,650 km^2^ and a population of 1,264,212 in 2014. There were 1208 basic schools and 26 high schools in 2004. In 2004, there were 5169 teachers in all schools, grades 1-9, and 656 teachers in high schools, grades 10-12 [67]. Solwezi has a land area of 125,826,656 km^2^, and a total population of 811,706 was recorded in 2014. Basic schools in 2012 were 747 and 23 high schools in 2004. Teachers in all schools in grades 1-9 were 2837 in 2004, while teachers in high schools in grades 10-12 were 509 the same year [67].

### Outcomes and Measures

Validated screening scales for self-reported symptoms will be used to assess clinical outcomes. These include the Mental Health Literacy Scale [69], the Generalized Anxiety Disorder 7-item (GAD-7) Scale (a GAD-7 score of ≥10 indicates moderate to high anxiety) [70], the Perceived Stress Scale (PSS-10) (a PSS-10 score of ≥10 indicates likely moderate to high stress) [71], the Brief Resilience Scale (BRS; BRS mean scores ranging from 1.00 to 2.99 indicate low resilience, scores from 3.00 to 4.30 suggest normal resilience, and mean scores from 4.31 to 5.00 suggest high resilience) [72,73], and the Patient Health Questionnaire-9 (PHQ-9; a score of ≥10 indicates moderate to high depression) [74]. Burnout will be assessed using the Maslach Burnout Inventory (MBI), specifically the MBI-Educators Survey, for use with educators [75,76]. An exploratory outcome will be to evaluate the acceptance of the Wellness4Teachers program by teachers in Zambia, which can be measured by the proportion of the target population (teachers in Livingstone, Lusaka, Kasama, and Solwezi) who subscribe to the program. Primary outcomes for this study will be changes in mental health literacy and the prevalence of moderate to high stress, burnout, moderate to high anxiety, moderate to high depression, and low resilience from baseline to 6 weeks, 3 months, 6 months, and 12 months among the subscribers of the Wellness4Teachers Zambia program. Secondary outcomes include the mean score on the Mental Health Literacy Scale and the prevalence of moderate to high stress, burnout, moderate to high anxiety, moderate to high depression, and low resilience at baseline in subscribers of the Wellness4Teachers email messaging program. Other secondary outcome measures will include changes in mean scores on the PSS-10, MBI, GAD-7, PHQ-9, and BRS. In addition, sociodemographic factors, work-related factors, and mental health literacy correlate with moderate to high stress, burnout, moderate to high anxiety, moderate to high depression, and low resilience at baseline among subscribers of the Wellness4Teachers program.

### Sample Size Estimation

With the total teacher population in Lusaka, Livingstone, Kasama, and Solwezi of about 30,561 [66], using a web-based script [77], it is projected that the sample size needed for our prevalence estimates with 95% confidence and a 3% margin of error for moderate to high stress, burnout, moderate to high anxiety, moderate to high depression, and low resilience among teachers in Livingstone, Lusaka, Kasama, and Solwezi will be 993. Based on the maximum survey completion rate attained for the Text4Hope and Text4Mood programs in Alberta of 20% [50,78,79], to achieve an estimated 993 completed surveys at baseline, it is planned to enroll at least 4965 teachers in the Wellness4Teachers email supportive and mental health literacy messaging program within 12 months.

### Statistical Analysis

SPSS (version 25; IBM Corp) [80] will be used to analyze the quantitative data from the surveys. Surveys with more than 50% missing responses will not be included in the data analysis. There will be no missing data imputation, and the analysis of the included survey responses and results will be based on completed survey data. Descriptive statistics for demographic, mental health literacy, clinical, and burnout-related variables will be provided. A 2-tailed *P*≤.05 will be used to determine statistical significance for all analyses, and descriptive characteristics will be presented as percentages and numbers. A chi-square test and logistic regression analysis will be used to separately identify demographic, clinical, and work-related correlates of anxiety, depression, stress, low resilience, and burnout for elementary and high school teachers. Significant and borderline significant variables from the chi-square analysis would be used to develop multivariable binary logistic regression models to predict the adjusted odds of outcomes for the exposure of interest and each potential correlate. The impact of the email supportive messaging program in reducing moderate to high anxiety, moderate to high depression, moderate to high stress, burnout, and improving resilience among subscribers will be evaluated by comparing the mean changes in these parameters from baseline to 6 weeks, 3 months to 6 months, and 12 months using the paired *t* test. To assess the effects of the intervention against a control group, we will choose a defined period of 3 months during the study period (for instance, the beginning of January 2023 to the end of March 2023) and compare the prevalence and mean scores on standardized scales for stress, anxiety, burnout, and depression at 6 weeks for subscribers who have received the daily supportive SMS text messages for 6 weeks (the intervention group) to the baseline prevalence and mean scores on the same scales for new subscribers during the period (the control group).

### Data Quality Assurance

At each stage of data collection, we will implement checks to ensure that we collect high-quality data, enabling us to generate accurate insights. A 3-member data quality committee will be established, which will be responsible for checking quantitative data that is uploaded to the server. All data will be checked for consistency and accuracy. Any errors and inconsistencies will be immediately communicated to the data collection team for corrective action.

### Ethics Approval and Informed Consent

The study has received ethics approval from the University of Alberta Ethics Review Board (Pro00117558), Dalhousie University Ethics Review Board 2022-6231, and Lusaka Apex Medical University, Zambia, Ethics Review Board IRB 00001131.00407-22. Participants’ consent to participate will be implied when they complete and submit the web-based survey responses. Participants will be informed that participation is voluntary, they can opt out anytime they desire, and the information they share is confidential. Teachers will not receive reimbursement or incentives for participating in the Wellness4Teachers email messaging program.

### Hypothesis

Based on the greater than 20% reduction in depression symptom scores in the intervention group compared to the control group in 2 randomized controlled trials in Ireland and Canada [56,57], and the greater than 20% reduction in anxiety symptom scores from baseline to 6 weeks and 3 months in subscribers of Text4Hope [51,52], we hypothesize that the Wellness4Teachers email messaging program will reduce the prevalence and severity of stress, burnout, anxiety, depression, and low resilience symptoms among Zambian teachers by at least 20%*.* We also hypothesize that the Wellness4Teachers email program will improve mental health literacy scores in the teachers subscribing to the program by at least 20%. In addition, we hypothesize that the prevalence of moderate to high stress, moderate to high anxiety, moderate to high depression, burnout, and low resilience would be comparable to the prevalence of these conditions reported in other jurisdictions [81,82]. Finally, we hypothesize that demographic factors, socioeconomic factors, organizational factors, and class size will be associated with burnout and other psychological disorders in teachers [16,19-23,49,58].

## Results

The Wellness4Teachers email messaging program is anticipated to be launched in January 2023. Enrollment will continue for 12 months, and data collection will continue for another 12 months. Results of the study will be disseminated to stakeholders in the education sector in Zambia, East African countries, and globally through workshops, conference presentations, and peer-reviewed publications.

## Discussion

### Principal Findings

Chronic stress can affect teachers, leading to burnout, anxiety, and depression. It can also indirectly affect students’ academic achievement since teachers’ performance is impacted by stress. In addition, this can also have a profound effect on teachers’ general lifestyle, physical health, psychological safety, and well-being, leading to reduced professional fulfillment and a low level of resilience. Teachers require innovative, convenient, easily accessible, and cost-effective programs to improve their mental health literacy and support their mental health due to their busy schedules. This protocol outlines the use of supportive email messages and mental health literacy e-health tools for implementing a psychological intervention for teachers who may be experiencing stress, burnout, depression, and anxiety and improving their overall resilience. Teachers with low mental health literacy, prior mental health conditions, and those exposed to previous traumas or adverse childhood experiences are likely to benefit substantially from this program.

Results from this study will provide key information about mental health literacy levels and prevalence rates of stress, burnout, depression, anxiety, and low resilience and their correlates among teachers in Zambia (Livingstone, Lusaka, Kasama, and Solwezi). Findings will also provide valuable knowledge on eHealth approaches in the education sector. Evidence of the effectiveness of daily supportive email messaging programs to address stress, burnout, anxiety, depression, and low resilience among teachers will be ascertained. Evidence from this study will be both essential and crucial for shaping school policy and decision-making regarding psychological interventions for teachers in low- and middle-income countries. It is anticipated that the outcomes of this study will provide an impetus for integrating supportive email and mental health literacy messaging interventions into many organizations’ occupational health programs.

### Limitations

Like most studies, this one has some limitations. First, the supportive email messages will be delivered for 12 months, and the outcome measures will be evaluated at 6 weeks, 3 months, 6 months, and 12 months. It is not certain what the effects of the intervention would be if it were extended. It is also unclear if the benefits of the intervention would diminish with the cessation of daily email messages. Second, although standardized, the self-reported scales used to assess mental health variables, such as moderate to high anxiety, are not diagnostic. Third, it is possible that participants’ demographics in the study may not reflect the demographics of the teacher population in Zambia; hence, the study findings may not be generalizable to all teachers in Zambia. In addition, web-based surveys with survey links delivered through SMS text messages usually achieve a response rate of less than 20% [54,78,79,83-86], so it is possible that we may not achieve our desired sample size with an email messaging program. Despite these limitations, this study is the first to assess the prevalence and correlates of stress, burnout, anxiety, and depression among teachers in Zambia using an email messaging program. To our knowledge, this study is also the first worldwide to assess if daily supportive email messages and mental health literacy information delivered by email would effectively reduce the prevalence and severity of psychological symptoms and improve mental health literacy among teachers.

### Conclusions

This study will provide vital information about mental health literacy and the prevalence and correlates of psychological problems among teachers in Zambia. The study will also provide evidence of the effectiveness of the email messaging program, Wellness4Teachers, in reducing psychological symptoms and improving mental health literacy among teachers. The knowledge from this study will significantly impact the promotion of mental health literacy and the management of stress, burnout, anxiety, depression, and low resilience among teachers. The study outcome will help inform policy decision-making for health care resource allocation in support of the educational sector in Zambia.

## Abbreviations

BRS: Brief Resilience Scale
GAD-7: Generalized Anxiety Disorder 7-item
MBI: Maslach Burnout Inventory
PHQ-9: Patient Health Questionnaire-9
PSS: Perceived Stress Scale

## Data Availability

The data sets that will be generated and analyzed during this study will be available from the corresponding author upon reasonable request.
